# The epidemiological characterization and geographic distribution of hepatitis D virus infection in Libya

**DOI:** 10.11604/pamj.2020.35.120.20055

**Published:** 2020-04-14

**Authors:** Mohamed Ali Daw, Amina Mohamed Daw, Nadia Emhemed Mohamed Sifennasr, Aisha Draha, Ahmed Daw, Ali Daw, Mohamed Ahmed, Ebtisam Mokhtar, Abdallah El-Bouzedi, Ibrahem Daw, Samia Adam, Saed Warrag

**Affiliations:** 1 Department of Medical Microbiology & Immunology, Faculty of Medicine, University of Tripoli, CC 82668, Tripoli, Libya; 2 Department of General Medicine, Faculty of Medicine, University of Tripoli, CC 82668, Tripoli, Libya; 3 Department of Pharmacology, Faculty of Medicine, University of Tripoli, CC 82668, Tripoli, Libya; 4 Tripoli Medical Centre, Faculty of Medicine, Tripoli, CC 82668, Tripoli, Libya; 5 Department of Microbiology & Parasitology, Faculty of Veterinary Medicine, University of Tripoli, CC 82668, Tripoli, Libya; 6 Department of Laboratory Medicine, Faculty of Biotechnology, Tripoli University, CC 82668, Tripoli, Libya; 7 Department of Electric Engineering, Faculty of Engineering, University of Tripoli, CC 82668Libya; 8 Department of Laboratory Medicine, Faculty of Biotechnology, Aljabel-Agarbi University, Nalot, Libya

**Keywords:** Libya, hepatitis D virus, risk factors

## Abstract

**Introduction:**

North Africa is known to be endemic for hepatitis D virus. However, data one the prevalence of this virus in Libya are scanty. This study aimed to determine the prevalence of hepatitis D virus infection in Libya and analyze the demographic factors associated with the infection, and also to assess the variations across the regions and districts.

**Methods:**

A total of 1873 samples collected from all over the country were tested for antibodies against hepatitis B surface antigen and the results were correlated with demographic and geographic variables.

**Results:**

The overall prevalence of hepatitis D virus infection was 1.7%. The prevalence rate was significantly high among those aged over 40 years (P < 0.001) and it was associated with intravenous drug use and coinfection with human immunodeficiency virus and/or hepatitis C virus infection (P < 0.001). The prevalence rates varied with geographic location and differed markedly within the regions the country. The highest rate reported was in the central region of Libya, followed by the western and eastern regions.

**Conclusion:**

Hepatitis D virus infection rate in Libya is considered to be low but is of some concern in some districts. This has been propagated by population displacement and African immigrants, indicating that a continuous epidemiological surveillance program should be implemented.

## Introduction

Hepatitis D virus (HDV) is an RNA viroid that infects only people in the presence of hepatitis B virus (HBV), which expresses hepatitis B envelope protein. HDV is responsible for the most severe form of acute and chronic viral hepatitis [1-4]. Worldwide, it is estimated that 2-8% of chronic HBV carriers are co-infected with HDV, corresponding to 10-20 million patients. Furthermore, HDV is transmitted by the same routes as HBV, including blood transfusion, sexual contact, intravenous drug abuse and parenteral transmission [5-7]. In the early 1980s, surveys showed that HDV was endemic worldwide but its prevalence rates are geographically diverse [8,9]. High prevalence rates of HDV have been reported in North African countries [10]. A recent meta-analysis showed that HDV is found in 5% of the general population in 20.7% of liver disease patients. The highest rates were reported in Egypt, Sudan and Mauritania, followed by Tunisia, Morocco, Algeria and Libya. The findings of that study should be interpreted with caution due to the small number of individuals tested, their demographic heterogeneity, and the quality of the testing methods used [11,12]. Libya has been plagued by a civil war since 2011, which has caused high rates of mortality, injury, population displacement, and major damage to the health care system at the structural and organizational levels. In addition, the breakdown of the security situation led to a heavy influx of African immigrants [13,14]. Together, these circumstances have maintained high levels of viral hepatitis transmission through injuries, blood and sexual contacts, and even unsafe blood transfusion [15]. Data on the impact of HDV in the country is even scarcer particularly as screening for anti HDV is not routine in HBsAg-positive patients. Therefore, it becomes important to reappraise the level of HDV infection among Libyan patients in order to plan a more effective preventive strategy. Hence, the objectives of this study were to determine the prevalence of HDV infection among Libyan patients, analyze the potential risk and demographic factors associated with the infection, and investigate the geographic variation of HDV within the different regions and districts of the country.

## Methods

Study population: the study involved 1873 HBsAg-positive serum samples that were collected from all Libyan regions and districts between 2015 and 2018. Appendix illustrates the geographic locations, regions, districts and population density. The national screening policy for HBV in Libya, which is uniform all over the country, includes screening of blood donors, preoperative patients, pregnant women and kidney and liver disease patients, as well as a mandatory pre-marriage screening. The study population included only those who were confirmed to be HBsAg-positive in the public health care system in Libya between 2015 and 2018, who were identified from the state wide data base with clear demographic and epidemiological information [11,12]. The data collected included region of origin, gender, age, level of education, marital status, and other related risk and demographic factors as previously described (12879_2013_2969_MOESM1_ESM.doc )[11].

Laboratory diagnosis: the study included all the samples that were HBsAg-positive and had detectable HBV DNA according to preliminary screening using “Real Time HBV Viral Load Assay” (Abbott Laboratories, IL). Anti-HDV antibodies in EDTA plasma samples were detected by using an enzyme-linked immunosorbent assay (ETI-AB-DELTAK-2, Diasorin, Saluggia, Italy) [11].

Statistical analysis: data were coded and entered into a database, which was then cleaned and verified [12]. Demographic and risk factors were compared between patients using chi-square tests or Fisher's exact test for categorical variables and Mann-Whitney U tests for continuous variables. Variables were considered statistically significant in the bivariate analysis when P < 0.001. All calculations were undertaken by using IBM SPSS Statistics for Windows, version 20.0 (IBM Corp., Armonk, NY).

Ethics approval and consent to participate: the study was approved by the Libyan National Ethical Committee (Approval No. LY NS, HDV-299-798-230). All participants signed an informed consent form witnessed by the local health office before collection of data and blood samples HIV [16,17].The questionnaire used to collect demographic and epidemiological data was anonymous and linked to the blood sample tube only by a code, as previously described [11].

## Results

Prevalence of Hepatitis D Virus: a total of 1911 patients with a national number (i.e. Libyan citizens) were initially included in the study but 38 (1.98%) were excluded due to lack of personal information. HBV testing was done uniformly. Of 1873 HBsAg-positive samples studied, 34 samples were positive for HDV, yielding an overall HDV prevalence of 1.7%. Of the 34 positive cases, 15 (44.1%) were females and 19 (55.9%) were males. No significant relationship between sex and HDV positivity was evident (P = 0.001). The mean age was 47.1 ± 13.4 years (median = 45.0). The highest HDV prevalence was among persons aged over 60 years (n = 13; 38.2%) followed by those aged 40-60 years (n = 11; 32.4%) and 21-40 years (n =7; 20.6%). It was lowest among those below 20 years of age (n = 3; 8.8%). The distribution of HDV seropositivity differed markedly from one region to another. The prevalence was higher in the central region (P < 0.001). Overall, HDV seropositivity was nearly twice more likely in the central region than in the other regions, and particularly compared to the eastern region.

Study population characteristics: Table 1 shows the demographic and risk factor features in the study population. A variety of risk factors were found to be significantly associated with HDV infection. HDV seropositivity was more prevalent among those with no formal education (41.2%, P < 0.001) than among those with primary or secondary level education. Furthermore, marital status, family history of HBV infection and sex were found to be less associated with HDV seroprevalence (P = 0.001). A higher prevalence was found among those who had a history of intravenous drug use (35.3%; P < 0.001), HCV infection (17.6%) or HIV infection (23.5%, P <0.001. On the other hand, only two patients had a history of blood transfusion.

**Table 1:** Demographic characteristics and risk factors among HDV-infected patients in Libya 2015-2018

Variables	HBV–HDV coinfection n (%)	HBV mono-infection n (%)	OR (95% CI)	P value
All Patients	34 (1.9)	1873 (94.3)		
Sex				
Males	19 (55.9)	1062 (56.7)	51.47 (22.4-87.36)	0.431
Females	15 (44.1)	811 (43.3)	39.71 (31.23-41.36)	0.211
Age Group				
0-20	3 (8.8)	437 (23.3)	4.21 (3.97-8.23)	0.15
21-40	7 (20.6)	553 (29.5)	11.23 (9.27-21.90)	0.311
40-60	11 (32.4)	512 (27.3)	27.10 (20.28-37.43)	< 0.001
>60	13 (38.2)	371 (19.8)	31.20 (27-43.23)	< 0.001
Region				
Western	9 (26.5)	536 (28.6)	21.01 (19.98-36.08)	0.021
Central	11 (32.4)	397 (21.2)	26.90 (20.01-38.10)	< 0.001
Southern	8 (23.5)	461 (24.6)	13.071 (12.91-19.04)	0.021
Eastern	6 (17.6)	479 (25.6)	7.12 (8.91-20.21)	0.054
Marital status				
Single	8 (23.5)	562 (30)	13.071 (12.91-19.04)	0.021
Married	10 (29.4)	763 (40.7)	24.11 (21.08-38.11)	0.311
Divorced	9 (26.5)	347 (18.5)	21.01 (19.98-36.08)	0.011
Unknown	7 (20.6)	201 (10.7)	11.23 (9.27-21.90)	0.051
Education				
No formal education	14 (41.2)	673 (35.9)	35.23 (29.12-40.31)	< 0.001
Primary	11 (32.4)	680 (36.3)	26.90 (20.01-38.10)	0.021
Secondary	9 (26.5)	520 (27.8)	21.01 (19.98-36.08)	0.210
Risk Factors				
Blood transfusion	2 (5.9)	221 (11.8)	2.31 (2.79-5.76)	
IVDU	12 (35.3)	276 (14.7)	28.71 (21.01-41.17)	
Coinfection				
HIV	8 (23.5)	317 (16.9)	13.071 (12.91-19.04)	< 0.001
HCV	6 (17.6)	113 (6.0)	7.12 (8.91-20.21)	< 0.001
Family HBV history	3 (8.8)	361 (19.3)	4.21 (3.97-8.23)	0.001
No risk factor known	3 (8.8)	585 (31.2)	4.21 (3.97-8.23)	0.001

OR (95% CI): odds ratio, 95% confidence interval.

**Figure 1:**
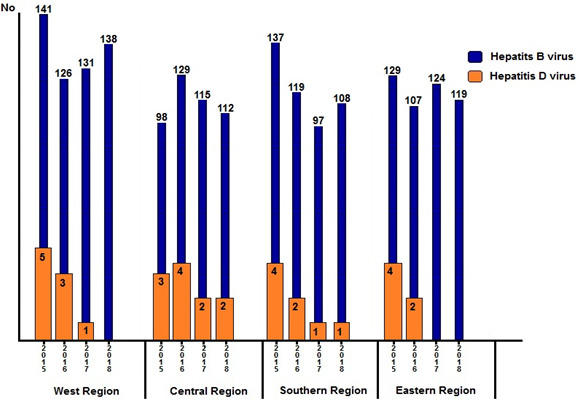
The annual number of cases of hepatitis D and hepatitis B infections in Libya 2015-2018

**Figure 2:**
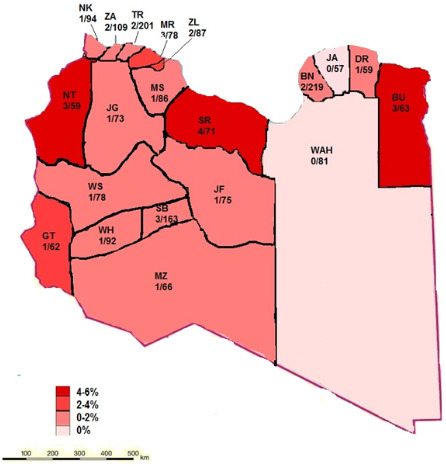
Geographic distribution of hepatitis D virus infections in Libya 2015-2018

Epidemiological patterns and geographic distribution: Figure 1 shows the annual frequencies of HBV and HDV diagnoses. The overall prevalence was higher in the central region (P < 0.001). In the western region, the highest rate was reported in 2015, after which it declined and reached zero in 2018. In the central region, the highest rates were in 2015 and 2016, after which the rate became steady in 2017 and 2018. In the southern region, the highest rate of HDV seropositivity was reported in 2015, after which it declined steadilyy until 2018. In the eastern region, HDV was reported only in 2015 and 2016. Figure 2 shows the geographic distribution of HDV in all Libyan districts. Seropositivity rates varied markedly from one district to another. Particularly high rates of sero-prevalence were reported in three districts. These include Sert (SR; 5.6%) in the central region, Nalout (NT; 5.1%) in the western region, and Butnan (BT; 4.8%) in the eastern region. These were followed by the districts of Margeb (MR) and Zletan (ZT) in the central region and Ghat (GT) in the southern region. The lowest seroprevalence rates were found in Murzak (MZ), Sebha (SB), Wadi Haiat (WH) and Wadi Shati (WS) in the southern region, followed by Jfara (JR), Musrata (MR) in the central region, and Tripoli, Zawia, Nuqat al Khams (NK) 1/94=1.1 and Ajfra in the western region. No HDV-positive cases were detected in Jabel Alkder (JK) and Wahat (WAH) in the eastern region.

## Discussion

In this large comprehensive nationwide study, we analyzed the sero-prevalence of HDV among 1873 HBsAg-positive individuals collected from all the regions and districts of the country during four years. Anti-Delta antibodies was observed in all age groups, with an overall prevalence of 1.7%. The highest rate was reported among those aged over 40 years and the lowest among those aged below 20 years. This 1.7% prevalence rate is very low compared with other Arab countries, such as Saudi Arabia (13.6% ), Mauritania (9-67%) and the neighboring countries Egypt (23.53%), Tunisia, (7-44% ) and Sudan (25-28%) [1,18,19].The prevalence of HDV infection in our cohort study was similar to the findings from other Mediterranean countries, including Spain and France, in which very low rates (<5%) were reported among chronic HBsAg-positive individuals [20,21]. This low rate of HDV infection in Libya, particularly among young adults, was expected because HBV vaccination has been strongly encouraged and offered free of charge in Libya since 1989 and it became compulsory for infants (3 months of age) and children (≥ 12 years of age). Furthermore, viral screening is mandatory in Libya for pregnant women, before surgical operations, and before a marriage contract is concluded. These measures have contributed immensely to reducing viral transmission within the Libyan population [11]. Notably, the use of highly effective anti-HBV vaccines has decreased both the prevalence and the spread of HBV and its associated pathogen, HDV. This has led to the belief that HDV eradication is approaching.

By analyzing the demographic characteristics contributing to the emergence of HDV in Libya, evidence of familial transmission, influence of sex and prior hospitalization were not found to be significant. Moreover, no particular risk factor was found to be associated with HDV co-infection. Injection drug use was the most probable route of HDV transmission for most of the patients in our study (P < 0.001). Those coinfected with HIV or HCV accounted for 23.5% and 17.6%, respectively. In contrast, in Europe, prevalence rates of HDV antibodies approaching 15% have been reported among HBsAg-positive carriers with HIV coinfection. 21 In Taiwan, a highly endemic region, incident HDV superinfections continue to occur. The prevalence rate of anti-HDV seropositive intravenous drug users among anti-HIV seronegative and anti-HIV seropositive cases was 40.0% and 84.2%, respectively [22]. The distribution of HDV diverged in the regions of the country and among distinct within the same region. HDV was reported in all Libyan districts, except for Jabel Alkder (JK) and Wahat (WAH). The central region had the highest prevalence of HDV. Of the 34 cases of detected in this study, 11 (32.4%) came from the central region, mainly from the districts of Sert, Murgab and Zleitan. In the central region, the highest rate was reported in 2016 and 2015, but it was steady in 2017 and 2018. The second region of a high HDV seropositivity was the western region, from where 26.5% of the cases came, and particularly from Nalut. The third region accounting for HDV prevalence was the southern region (23.5%), where there was a uniform distribution of HD cases among the districts, apart from Ghat. The eastern region had the lowest rate of HDV (17.6%), with most of the cases located mainly in Butnan, neighboring Egypt.

The observed geographic variation in the prevalence of HDV in this study could be related to “micro-epidemiology” in different areas of the country. Large regional and district variation in the prevalence of HDV has previously been reported in other African countries, such as Ethiopia. Aberra *et al.* (2018) have shown that certain geographical areas in Ethiopia, such as Amhara, Addis Ababa and Afar, appeared to have a higher HDV prevalence than the rates reported in Oromia, SNNPR and Tigray, though the overall prevalence in Ethiopia was reported to be low [23]. In our study, there was a significantly higher prevalence of HDV infection in certain Libyan districts, particularly in the central and western regions. However, the numbers were small and these observations need confirmation in a population-based survey and further investigation are needed to reveal whether specific genetic or cultural factors influence HDV transmission [24-26]. Libya has experienced a major population shifts in 2011. Over 4% of the population were displaced due to internal conflict and a major exodus of African immigrants who had been residing towards the European Union countries [27-29]. Studies on viral hepatitis among African immigrants in Libya have shown that the prevalence of HBV ranged from 8 to 25%, depending on the country of origin. Hence, surveillance of HDV, universal access to HBV vaccination, and improvements in socioeconomic and educational status for both local citizens and immigrants remain the keystone for HDV control strategies [30-32].

## Conclusion

This work represents the first large-scale countrywide analysis of the sero-prevalence of HDV in Libya, which is one of the largest countries in North Africa. However, the unselected nature of our study population, in which all patients were screened for HBV and HDV infection independently of clinical manifestations or laboratory abnormalities, might have played a role in generating false positive results [33,34]. Our study shows a current very low rate of HDV infection among chronic HBsAg-positive individuals in Libya. This prevalence is diverse among regions and districts, displaying the highest rates in the central region. However, this study did not evaluate the HBV load within the studied population, which might have underestimated the true HDV prevalence. Further studies are needed to analyze the genetic diversity of HDV, and a screening policy for HDV should be implemented, at least among patients with hepatic liver diseases, immigrants, and groups at a higher risk [35-39].

### What is known about this topic

- This study is a national surveillance study carried on HDV infection in Libya, the second largest Country in Africa;
- The prevalence of HDV varies geographically and over time within the Libyan regions and districts;
- Different demographic factors were found to contribute to the prevalence of HDV in Libya.

### What this study adds

- Libya in low endemic country regarding HDV infection;
- HIV and IVDUs are the main contributing factors in HDV in Libya;
- Hepatitis D virus screening should be implanted in Libya.

## Competing interests

The authors declare no competing interests.
